# The Theory of Planned Behavior and Antecedents of Attitude toward Bee Propolis Products Using a Structural Equation Model

**DOI:** 10.3390/foods13183002

**Published:** 2024-09-22

**Authors:** Kyung-A Sun, Joonho Moon

**Affiliations:** 1 Department of Tourism Management, Gachon University, Sungnam-si 13120, Republic of Korea; kasun@gachon.ac.kr; 2 Department of Tourism Administration, Kangwon National University, Chuncheon 24341, Republic of Korea

**Keywords:** bee propolis products, attitude, theory of planned behavior, price fairness, healthiness, eco-friendliness, ease of use

## Abstract

This work examines consumers’ perceptions of products containing bee propolis using the theory of planned behavior as a theoretical foundation. As antecedents of attitude, this work employs price fairness, healthiness, eco-friendliness, and ease of use. A survey was issued to participants who had experience using bee propolis products and who were recruited using the Clickworker platform service. In total, 305 valid observations were collected for analysis. This study used a maximum likelihood-based structural equation model to test the research hypotheses and find that price fairness, healthiness, eco-friendliness, and ease of use positively affected attitude. Moreover, the intention to use is positively impacted by attitude, subjective norms, and behavioral control. This research contributes to the literature by demonstrating the explanatory power of the theory of planned behavior with respect to bee propolis products.

## 1. Introduction

Bee propolis products (BPPs) in the United States had a market size of approximately 636 million US dollars in 2021, and these products are projected to achieve a market growth rate of 2.8% between 2022 and 2028 [1]. Bee propolis is commonly used for therapeutic purposes [2] and may promote health via its antioxidant effects and ability to minimize the risk of cardiovascular disease [3,4]. Grand View Research [1] also stated that BPP is attractive to consumers because consumers value their health more, and BPP can meet such needs because it has preventive and remedy functions. Thus, bee propolis has the potential to become a particularly lucrative commodity. Under this condition, it is essential to understand consumer characteristics because such information could become the starting point for the market growth of BPPs. However, consumer behavior concerning this product remains largely unexplored. 

This research employs the theory of planned behavior, which has been adopted in various domains to investigate consumer behavior [5,6,7,8], as the theoretical foundation. The main attributes of this work include attitude, subjective norm, and behavioral control in the theory of planned behavior, and the dependent variable is the intention to use, in line with the previous works [9,10]. Although numerous studies demonstrated the explanatory power of the theory of planned behavior in the consumer behavior domain [11,12,13], consumers’ behavior in the case of BPPs has been sparsely explored using the theory of planned behavior as the theoretical foundation. Considering such a research gap, this research tests the explanatory power of the theory of planned behavior in the case of BPPs. 

Attitude is the long-term appraisal of goods and services [5,8,14]. The merits of BPPs are likely to include their affordability [15], indicating that price is likely to become the strength of BPPs. Scholars also argued that healthy properties are the motivation for BPP consumption because it improves an individual’s immune system [16,17]. Moreover, prior works noted that eco-friendliness is an appealing point of BPPs because consumers are skeptical of chemical-based medicine [18,19]. Last, researchers claimed that ease of use is an important aspect of consumer choice because complexity causes negative consequences in the area of consumer behavior [20,21]. All things considered, this research employs four attributes to account for the attitude of consumers in the area of BPPs. 

In summary, the first objective of this work is to test the explanatory power of the theory of planned behavior in the area of BPPs. The second objective of this research is to investigate the antecedents of attitude, which is a main variable in the theory of planned behavior. As antecedents, this research proposes four attributes: price fairness, healthiness, eco-friendliness, and ease of use. Even though numerous studies have explored BPP characteristics [16,22,23], researchers have insufficiently examined the consumer perception of BPPs. Such a research gap leads this study to inspect consumer perception of BPPs using the theory of planned behavior as a theoretical foundation. Also, this research sheds light on the literature by scrutinizing the determinants of attitude in BPPs. Additionally, this research discusses managerial implications. 

## 2. Review of Literature and Hypothesis Development

### 2.1. Theory of Planned Behavior

The theory of planned behavior discusses the determinants of intention, including attitude, subjective norms, and behavioral control [7,14]. Attitude is the individual appraisal of a subject based on a long time [6,24]; subjective norms constitute others’ expectations of and perspectives on certain behaviors that create social pressure [5,8]. Behavioral control relates to constraints regarding available resources [12,13]. Many studies have used the theory of planned behavior in diverse domains. For instance, Tama et al. [11] explored farmers’ intentions regarding the conservation of agriculture using this theory as a theoretical background; Fan et al. [6] used it to examine student behavior concerning vaccinations in China; Ateş [5] investigated the antecedents of pro-environmental behavioral intentions using the theory as the framework; Conner et al. [25] applied it to investigate healthy eating habits; and Chen [26] and Lim and An [27] adopted it as a theoretical foundation to account for consumers’ healthier food choices. 

### 2.2. Hypothesis Development and Consumer Research on BPPs

The price of BPPs may support a positive consumer perception of them on the market, and consumers are likely to perceive the price reasonably [15]. Still, their health-promoting properties [16,17], eco-friendliness (they are largely free from chemical contaminants) [18,19], and ease of use (being available in pill, spray, and gel forms) [20,21] are also key strengths. This research thus examines the determinants of attitude using four attributes: price fairness, healthiness, eco-friendliness, and ease of use. 

The first area is price fairness. Price fairness refers to how rationally consumers assess the listed price of the seller [28,29]. Price rationality plays a significant role in persuading consumers to purchase a product [28,30]. Rai [29] demonstrated a positive effect of price fairness on attitude. Sohaib et al. [31] examined well-being products that consumers favor and detected a positive association between price fairness and attitude. Syah et al. [15] explored consumers in Brazil, and the findings implied that price significantly affected consumer perception. Such findings are likely to be applied to the case of BPPs because BPPs aim to promote the well-being of consumers. 

Perceived healthiness plays a significant role in building positive attitudes and promoting purchase intention [32,33]. Jang et al. [34] found that healthiness positively impacted consumer attitudes toward in-flight meals, while Janssen and Bogaert [35] demonstrated its positive effects based on food packaging information. El-Sakhawy et al. [23] additionally alleged that consumers value BPPs more because they promote individuals’ health condition by strengthening the immune system. Based on the review of the literature, the following research hypotheses are proposed:

**Hypothesis 1:**  *Price fairness positively affects attitude.*

**Hypothesis 2:**  *Healthiness positively affects attitude.*

Eco-friendliness refers to a product’s environmental impact in terms of its ingredients and the minimization of harmful effects on the environment [36,37]. Given that consumers generally perceive eco-friendly products as safer to eat [37,38], they are more likely to develop positive attitudes toward them. A meta-analysis found that eco-friendliness helps determine attitude [39]. Other studies have reported a positive influence of eco-friendliness on consumer attitude [36,38] because eco-friendly products can make consumers perceive food products more safely. Additionally, Mountford-McAuley et al. [40] suggested that the environmental aspect of BPPs offers a key marketing opportunity, as consumers place greater importance on environmental attributes, particularly in the context of medical products. 

Regarding ease of use, which refers to the simplicity with which goods may be used by consumers [41,42], consumers tend to avoid products that they perceive as complex and confusing or that require time and effort to use [43,44]. They may perceive such goods as requiring an extra investment to learn how to use the products, which functions as a deterrent. Previous research has demonstrated a positive association between ease of use and attitude in various areas: e-commerce [45], smartphone chatbots [41], augmented reality [44], and online learning [43]. In the case of functional food such as BPPs, complexity causes negative perception because misuse is likely to be harmful to health conditions. Additionally, Grand View Research [1] also noted that BPPs are likely to be used in various types: ointment, spray, and pill for the convenience of consumers. Hence, we propose the following:

**Hypothesis 3:**  *Eco-friendliness positively affects attitude.*

**Hypothesis 4:**  *Ease of use positively affects attitude.*

Several previous studies have considered the intention to use the consumer behavior domain in the framework of the theory of planned behavior [46,47]. For example, a meta-analysis revealed that attitude, subjective norms, and behavioral control positively affect individual intention [7], with studies showing the positive influences of these on farmers’ intentions regarding conservation agriculture [11], intention to use the Alipay e-wallet system [10], and intention to use electronic vehicles [9]. It can be inferred that the attributes in the theory of planned behavior have played a significant role in explaining consumer intention to use. Thus, we propose the following to ensure its accountability in the domain of BPPs:

**Hypothesis 5:**  *Attitude positively affects intention to use.*

**Hypothesis 6:**  *Subjective norms positively affect intention to use.*

**Hypothesis 7:**  *Behavioral control positively affects intention to use.*

## 3. Method

### 3.1. Research Model

**Figure 1:**
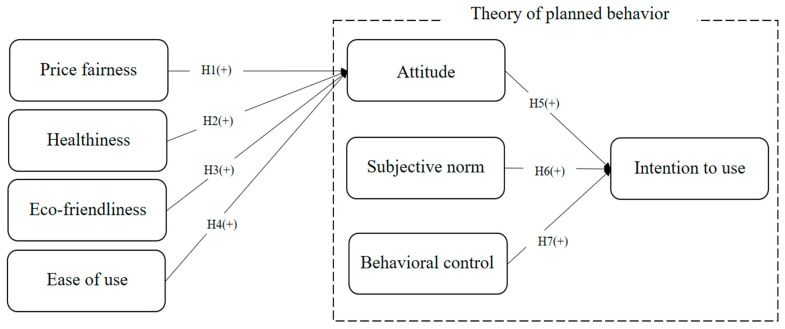
Research model.

Figure 1 illustrates the research model, which includes eight attributes: price fairness, healthiness, eco-friendliness, ease of use, attitude, subjective norms, behavioral control, and intention to use. Price fairness, healthiness, eco-friendliness, and ease of use are the determinants of attitude, all of which have a positive impact on attitude. Moreover, intention to use is positively influenced by attitude, subjective norms, and behavioral control. 

### 3.2. Description of Measurement Items

Table 1 presents the measurement items. A Likert five-point scale (1 = strongly disagree, 5 = strongly agree) was used to measure most attributes, while attitude was measured using a semantic differential scale (e.g., 1 = bad, 5 = good). Price fairness is defined as consumers’ assessment of the price of BPPs as being rational. We pull these items from prior studies but modify them for greater applicability to this present study. The main attributes are price fairness [28,31], healthiness [32,34], eco-friendliness [34,36], ease of use [41,44], attitude [48,49], subjective norm [12,13], behavioral control [5,6], and intention to use [9,10,50]. This research defined price fairness as how consumers perceived the price of BPPs. The definitions are given above. The operational definition of healthiness is how consumers perceive the BPPs to promote health conditions. Eco-friendliness is defined as how the BPPs are related to the environmental aspects. Ease of use is defined as how BPP is simple to use. Attitude is defined as a long-term evaluation of BPPs. The definition of the subjective norm is a sort of popularity of BPPs from people. Behavioral control is defined as whether an individual is disturbed by the consumption of BPPs. Last, the operational definition of intention to use is how willing individuals are to purchase BPPs.

### 3.3. Data Collection 

The Clickworker (https://www.clickworker.com/, accessed on 11 June 2024) platform was used to recruit survey participants. Native English speakers were targeted and the data were collected between 23 June and 2 August 2024. Participants were asked whether they had experience using BPPs, and 650 responses were initially collected. Those with no experience using BPPs (345 respondents) were eliminated from the dataset because this research targeted the responses from the vivid BPP experience. Consequently, 305 observations remained for analysis. Table 2 details the survey participants’ profiles. 

### 3.4. Data Analysis 

Frequency analysis was used to derive the survey participants’ demographic information. Confirmatory factor analysis was performed to examine convergent validity. The convergent validity of measurements was ensured by multiple indices: loading > 0.5, average value extracted (AVE) > 0.5, and construct reliability (CR) > 0.7 [51,52]. The goodness-of-fit was confirmed using multiple indices: Q (CMIN/degrees of freedom) < 3, the goodness-of-fit index (GFI), the normed fit index (NFI), the relative fit index (RFI), the comparative fit index (CFI) > 0.8, and the root mean square error of approximation (RMSEA) < 0.1 [52,53]. Then, the mean values and standard deviations (SDs) were computed for the variables. The correlation matrix was adopted not only to explore the relationships between attributes but also to ensure discriminant validity. The square root of AVE should be greater than the correlation coefficient for an acceptable discriminant validity level [51,52,53]. A maximum likelihood-based structural equation model was further conducted to test hypotheses using a confidence interval of 90 percent as the threshold.

## 4. Results 

### 4.1. Convergent Validity and Discriminant Validity 

Table 3 presents the results of the confirmatory factor analysis. The goodness-of-fit indices indicate that the results are statistically acceptable (χ^2^ = 912.863, df = 442, χ^2^/df = 2.065, GFI = 0.837, NFI = 0.899, RFI = 0.886, CFI = 0.945, and RMSEA = 0.059). All factor loadings, AVEs, and CRs were greater than the cut-off value, suggesting that the convergent validity of the measurement items was adequate.

Table 4 presents the correlation matrix. The diagonal values are greater than the off-diagonal values, indicating that the discriminant validity of the measurement items was appropriate. Intention to use is positively correlated with attitude, subjective norm, behavioral control, price fairness, healthiness, ease of use, and eco-friendliness. Attitude also positively correlates with subjective norms, behavioral control, price fairness, healthiness, ease of use, and eco-friendliness.

### 4.2. Hypotheses Testing Using Structural Equation Model 

Table 5 details the results of the hypothesis testing. Attitude is positively affected by price fairness, healthiness, eco-friendliness, and ease of use. Intention to use is also positively influenced by attitude, subjective norm, and behavioral control. Thus, all hypotheses are supported.

## 5. Discussion

The first objective of this study was to evaluate the explanatory power of the theory of planned behavior in the context of BPPs. To achieve this, the research focused on four key attributes: attitude, subjective norms, perceived behavioral control, and intention to use. Specifically, the theory of planned behavior was used to assess consumer behavior related to BPPs. Our findings revealed that attitude, subjective norms, and perceived behavioral control had positive effects on the intention to use BPPs. In addition, the results indicated that subjective norm and behavioral control positively affected the intention to use BPPs. Furthermore, attitude showed the strongest impact on the intention to use BPPs compared to subjective norms and behavioral control. The findings of this work are aligned with Conner et al. [25]’s argument that the theory of planned behavior could become a key framework for health-related research areas. 

The second objective was to explore the determinants of consumer attitudes toward BPPs. The results are significant in identifying strong correlations between attitude and four key factors: price fairness, healthiness, eco-friendliness, and ease of use. Notably, price fairness positively influenced consumer attitudes, suggesting that BPPs’ pricing enhances their market appeal. Namely, the price is an imperative attribute for consumer appraisal, as scholars claimed [28,29]. Additionally, healthiness, eco-friendliness, and ease of use played crucial roles in shaping positive attitudes toward BPPs, indicating that these attributes should be emphasized in marketing strategies. In other words, consumers developed favorable attitudes toward BPPs because they perceived them as promoting personal health, minimizing environmental harm, and being easy to use. Among these factors, healthiness had the strongest impact. Because BPPs aim to promote health by strengthening the immune system, these findings may align with the main motivators underpinning BPP consumption from the extant literature [16,22,23]. Moreover, it can be inferred that the environmental aspect has become more important for appraisals of consumers in the food market, as prior works stated [40]. Furthermore, this research demonstrated the importance of ease of use in the case of functional food by following the reasons that complexity causes cost from the perspective of consumers [43,44]. 

## 6. Conclusions

The key theoretical contributions are as follows. First, this research demonstrated the applicability of the theory of planned behavior in the area of BPPs. Although numerous studies have addressed the characteristics of BPPs [16,22,23], few have explored consumer perceptions of BPPs using the theory of planned behavior. Conner et al. [25] highlighted that the theory of planned behavior primarily focuses on healthy eating, suggesting that it is a suitable theoretical framework for explaining consumer behavior related to BPPs. This present study addressed this gap by validating the theory’s explanatory power and identifying the key determinants of consumer attitudes toward BPPs. Our results demonstrated the significant effects of price fairness, healthiness, eco-friendliness, and ease of use in consumer research. These findings contribute to the literature by providing a deeper understanding of consumer characteristics in the context of BPPs. Specifically, the results align with existing studies by highlighting the significant influence of price [15] and healthiness [34] in the food product sector. Additionally, they support the findings of previous research, suggesting that eco-friendliness is a key factor in consumer marketing [39]. It can be inferred that eco-friendliness has become increasingly important in consumer behavior due to a higher interest in environmental issues such as global warming. Moreover, the findings reinforce the importance of ease of use, particularly in the functional food domain [44,45]. 

Several managerial implications emerged. Above all, managers may need to adopt a more conservative approach to price changes to avoid undermining consumers’ perceptions of price fairness because varied prices are likely to undermine price fairness perceptions. Additionally, managers may need to implement a price comparison system to provide consumers with greater transparency. Investing in healthier ingredients is also crucial, as healthiness has emerged as a key motivator. Managers could further appeal to consumers by making nutrition labels more visible. Emphasizing both the environmental benefits and ease of use of BPPs is important for fostering positive consumer attitudes. Specifically, marketing messages should highlight information on price, health-related ingredients, eco-friendly features, and product simplicity, as these factors are strongly associated with positive attitudes. Managers may also consider incorporating visuals or messages related to healthiness and eco-friendliness, which are likely to enhance consumer attitudes. Moreover, allocating resources toward convenient product designs, such as ointments, pills, and sprays, could improve the sales of BPPs, given that ease of use is a strong driver of purchase decisions. In addition, resources should be invested in fostering more positive attitudes, given that attitude functions as the antecedent of intention to use. It may also be worthwhile to focus more on peer group marketing, identifying a target market of consumers who possess sufficient resources to consume BPPs. This could ultimately increase the sales of BPPs. Managers also might need to contemplate that focusing on the healthiness-related area for marketing could become the most efficient resource allocation because it shows the strongest effect as compared to the other three attributes. 

This research had several limitations that should be mentioned. First, we focused exclusively on the linear effect between attributes, and future research should consider more diverse relationships, such as moderating effects and curvilinear impacts, to further elucidate consumer behavior. Moreover, a survey was used as the sole data-collection instrument; future studies might consider a broader range of methods such as an experimental design. In addition, the study participants were exclusively native English speakers; future works may consider more diverse geographic cases, given that food and nutrients may be perceived differently in various cultural contexts and under the influence of market trends. This research has another limitation because the survey participants were experienced with the BPPs. Future research might be able to consider respondents without experience with BPPs because the outcome might yield further information for the potential market. 

## Figures and Tables

**Table 1:** Description of measurements.

Construct	Code	Item
Price fairness	PF1	The price of BPPs is fair.
	PF2	The price of BPPs is reasonable.
	PF3	The price of BPPs is rational.
	PF4	The price of BPPs is acceptable.
Healthiness	HE1	BPPs support my good health.
	HE2	BPPs are useful for better health.
	HE3	BPPs improve my health condition.
	HE4	BPPs are effective for enhancing my health.
Eco-friendliness	EF1	BPPs are eco-friendly.
	EF2	BPPs are environmentally friendly.
	EF3	BPPs do not cause ecological harm.
	EF4	BPPs are environmentally safe.
Ease of use	EU1	BPPs are easy to use.
	EU2	BPPs are simple to use.
	EU3	BPPs are straightforward to use.
	EU4	It is not complex to use BPPs.
Attitude	AT1	For me, BPPs are (bad/good).
	AT2	For me, BPPs are (negative/positive).
	AT3	For me, BPPs are (unfavorable/favorable).
	AT4	For me, BPPs are (foolish/wise).
Subjective norm	SN1	People around me seem to consider the use of BPPs naturally.
	SN2	People around me believe that BPPs represent ethical consumption.
	SN3	People close to me believe that the consumption of BPPs is easy.
	SN4	People who are important to me consider it possible to use BPPs.
Behavioral control	BC1	I have enough resources to buy BPPs.
	BC2	I have enough money to buy BPPs.
	BC3	There are no obstacles to my use of BPPs.
	BC4	I have sufficient resources to purchase BPPs.
Intention to use	IU1	I intend to use BPPs.
	IU2	I will purchase BPPs.
	IU3	I am willing to buy BPPs.
	IU4	I have an intention to use BPPs.

**Table 2:** Profiles of survey participants (N = 305).

Item	Frequency	Percentage
Male	126	41.3
Female	179	58.7
20–29 years	47	24.3
30–39 years	101	33.1
40–49 years	92	30.2
50–59 years	33	10.8
Older than 60 years	5	1.6
Monthly household income		
Less than USD 2500	86	28.2
Between USD 2500 and USD 4999	111	36.4
Between USD 5000 and USD 7499	48	15.7
Between USD 7500 and USD 9999	13	4.3
More than USD 10,000	47	15.4
Weekly use frequency		
None	118	38.7
One to two times	121	39.7
Three to six times	44	14.4
More than seven times	22	7.2

**Table 3:** Confirmatory factor analysis results.

Construct	Code	Loading	Mean (SD)	AVE	CR
Price fairness	PF1 PF2 PF3 PF4	0.860 0.890 0.845 0.807	3.57 (0.84)	0.724	0.919
Healthiness	HE1 HE2 HE3 HE4	0.885 0.868 0.863 0.832	3.84 (0.86)	0.743	0.920
Eco-friendliness	EF1 EF2 EF3 EF4	0.882 0.817 0.895 0.864	3.92 (0.84)	0.748	0.922
Ease of use	EU1 EU2 EU3 EU4	0.773 0.871 0.893 0.885	4.19 (0.76)	0.734	0.916
Attitude	AT1 AT2 AT3 AT4	0.841 0.831 0.881 0.695	4.00 (0.74)	0.664	0.887
Subjective norm	SN1 SN2 SN3 SN4	0.830 0.845 0.843 0.799	3.55 (0.90)	0.687	0.898
Behavioral control	BC1 BC2 BC3 BC4	0.798 0.827 0.823 0.881	3.66 (0.94)	0.693	0.900
Intention to use	IU1 IU2 IU3 IU4	0.927 0.936 0.896 0.899	3.84 (1.01)	0.833	0.953

Note: SD, standard deviation, goodness-of-fit indices: χ^2^ = 912.863, df = 442, χ^2^/df = 2.065 GFI = 0.837; NFI = 0.899; RFI = 0.886; CFI = 0.945; RMSEA = 0.059; CR, construct reliability; AVE, average variance extracted.

**Table 4:** Correlation matrix.

	1	2	3	4	5	6	7	8
1. Intention to use	0.914							
2. Attitude	0.656 *	0.814						
3. Subjective norm	0.574 *	0.490 *	0.828					
4. Behavioral control	0.432 *	0.365 *	0.499 *	0.832				
5. Price fairness	0.468 *	0.478 *	0.560 *	0.461 *	0.850			
6. Healthiness	0.684 *	0.631 *	0.562 *	0.354 *	0.435 *	0.861		
7. Ease of use	0.533 *	0.505 *	0.416 *	0.467 *	0.350 *	0.493 *	0.856	
8. Eco-friendliness	0.655 *	0.579 *	0.570 *	0.386 *	0.505 *	0.584 *	0.493 *	0.864

Note: * *p* < 0.05; diagonal is the square root of average variance extracted; SD, standard deviation.

**Table 5:** Results of hypotheses testing.

Path	β	Critical Ratio	*p* Value	Results
Price fairness → Attitude	0.132 **	2.72	0.006	H1 supported
Healthiness → Attitude	0.345 **	6.37	0.000	H2 supported
Eco-friendliness → Attitude	0.170 **	3.01	0.003	H3 supported
Ease of use → Attitude	0.168 **	2.97	0.003	H4 supported
Attitude → Intention to use	0.787 **	9.58	0.000	H5 supported
Subjective norm → Intention to use	0.325 **	4.87	0.000	H6 supported
Behavioral control → Intention to use	0.106 *	1.75	0.079	H7 marginally supported

Note: * *p* < 0.1, ** *p* < 0.05.

## Data Availability

The data presented in this study are available upon request from the corresponding author. The data are not publicly available due to privacy.
